# Hoffa fractures are associated with concomitant soft tissue injures and a high postoperative complication rate

**DOI:** 10.1007/s00402-023-05133-0

**Published:** 2023-12-13

**Authors:** M. V. Neumann-Langen, L. Eggeling, R. Glaab, F. von Rehlingen-Prinz, C. Kösters, E. Herbst

**Affiliations:** 1 Department of Orthopaedic Trauma Surgery, Klinikum Konstanz, Mainaustrasse 35, 78464 Constance, Germany; 2 Department of Orthopaedic and Trauma Surgery, University Hospital of Freiburg, Hugstetter Strasse 55, 79104 Freiburg, Germany; 3 Department of Trauma and Orthopaedic Surgery, Sports Traumatology, BG Klinikum Hamburg, Bergedorfer Strasse 10, 21033 Hamburg, Germany; 4 Department of Orthopaedics and Traumatology, Cantonal Hospital Aarau, Tellstrasse 25, 5001 Aarau, Switzerland; 5 Department of Orthopaedic and Trauma Surgery, Universitätsklinikum Hamburg-Eppendorf, Gebäude O10, Martinistrasse 52, 20246 Hamburg, Germany; 6 Department of Orthopaedic and Trauma Surgery, Maria-Josef-Hospital Greven, Lindenstrasse 29, 48268 Greven, Germany; 7 Department of Orthopaedic and Trauma Surgery, University Hospital of Muenster, Albert-Schweitzer-Campus 1, 48149 Münster, Germany

**Keywords:** Knee, Hoffa, Fracture, Complication, Outcome

## Abstract

**Introduction:**

Hoffa fractures are a rare and often overlooked entity. The main goal of surgical treatment is to restore the articular surface and maintain knee function. However, current clinical data indicate heterogeneous outcomes. The aim of this multicenter study was to obtain a representative data set of patients with isolated Hoffa fractures with special emphasis on concomitant soft tissue injuries, diagnostic algorithms, treatment strategies and functional outcomes.

**Materials and methods:**

Participating Level I trauma centres were asked to review their internal database for isolated Hoffa fractures treated surgically between 2010 and 2020. Demographics, mechanism of injury, diagnostic and therapeutic algorithm, Letenneur classification, concomitant soft tissue injuries, and postoperative knee function and complications were analysed.

**Results:**

A total of 56 patients from six participating trauma centres were included. The median age at injury was 45 years (15–94) with a median follow-up of 19 months (2–108). The most common mechanism of injury was high-energy trauma, with unicondylar lateral Letenneur type I and II fractures being the most common. Surgical treatment was independent of the type of fracture and included isolated screw fixation, combined plate and screw fixation and isolated plate osteosynthesis. Isolated screw fixation resulted in significantly better range of motion (ROM) values (*p* = 0.032), but the highest number of postoperative complications (*n* = 14/20, n.s.) compared to the other fixation techniques. The highest number of fixation failures requiring revision was observed in the plate and screw fixation group (*n* = 3/8, *p* = 0.008). Osteochondral flake fractures (*n* = 12/43, 27%) and lateral meniscus injuries (*n* = 5/49, 10%) were commonly seen in Hoffa fractures.

**Conclusions:**

Treatment of Hoffa fractures with screw fixation resulted in significantly better functional outcomes, probably due to less comminuted fractures. Concomitant cartilage, meniscal and ligamentous injuries are common and warrant preoperative recognition and management.

## Introduction

Hoffa fractures defined as coronal plane shear fractures of the femoral condyle are rare but devastating injuries, accounting for 8.9–13% of all distal femur fractures [1–4].

Hoffa fractures are more common in young individuals and occur because of high-energy trauma involving knee flexion of ≥ 90° and an axial force in either varus or valgus direction [5, 6]. Due to the physiological genu valgum of the knee joint, the lateral condyle is more commonly injured [7–9]. In contrast to these high-energy injuries, low-energy trauma resulting in Hoffa fractures is seen in patients with skeletal immaturity [10], or in elderly subjects with osteoporosis [11].

Regardless of the degree of fracture displacement, open reduction and internal fixation remains the preferred method of treatment for Hoffa fractures to anatomically restore the articular surface [12, 13]. In general, surgical fixation of these fractures depends on the fracture pattern and the size of the condylar fragments. Most commonly, osteosynthesis with screws is performed, with however variable clinical and biomechanical results [14, 15]. Plate fixation, chosen as an antiglide plate can provide biomechanically more stable constructs [16–18], especially in revision cases. Hybrid fixation methods using combined techniques of screws and a plate are recommended in patients with poor bone quality, metaphyseal fracture extension or comminuted Hoffa fractures [19, 20]. However, there is no general consensus on the diagnostic approach and treatment strategy for Hoffa fractures.

Therefore, the aim of this study was to retrospectively analyse the current standard of care for Hoffa fractures in six level I trauma centres in Germany and Switzerland. Fracture patterns, concomitant soft tissue injuries and functional outcomes were also recorded.

## Materials and methods

Participating Level I trauma centres were asked to retrospectively review their internal database for isolated Hoffa fractures surgically treated between January 1, 2010 and December 31, 2020. To facilitate data retrieval, the International Classification of Diseases (ICD), 10th Revision variable S72.44 for distal femoral fractures was used. Radiographic data of the screened subjects were then analysed to identify coronal plane fractures of the distal femur. Exclusion criteria were fractures in subjects with open physes and periprosthetic fractures. Data collected included patient sex and age at surgery, trauma mechanism (high or low energy), the performed preoperative radiographic procedures and fracture classification using the AO alphanumeric classification system and the Letenneur classification [21].

Surgical treatment strategy including the surgical approach and fixation method (screw vs plate vs combined techniques) was documented. Preoperative and intraoperative findings regarding additional knee injuries, such as ligamentous, meniscal or cartilage lesions, were recorded. The incidence of postoperative complications including impaired wound healing, surgical-site infection, loss of reduction with subsequent fixation failure and delayed bone union was documented. Total follow-up and the range of motion (ROM) of the knee joint at final follow-up were also recorded.

Statistical analysis was performed using OriginPro, version 2023, OriginLab Corporation, Northampton, MA, USA. Due to the non-normal distribution of the values, a non-parametric test was used. For continuous variables, Kruskal–Wallis test with Dunn’s test for comparison of more than two groups was used. For ordinal data, the chi-square test was used. A *p* value < 0.05 was considered statistically significant. Due to the exploratory nature of the study, no correction for multiple testing was made.

The study protocol was approved by the local Human Research Ethics Committee (EKF 22-1287-S1-retro).

## Results

A total of six level I trauma centres participated in the study, with 56 patients meeting the inclusion criteria. The patient population consisted of 34 (60.7%) male and 22 (39.3%) female patients with an average age of 45 years (range: 15–94 years) at the time of surgery. Patient demographics are shown in Table 1.

**Table 1:** Overview of demographic patient data

	Plate and screw fixation	Screw fixation only	Plate fixation only	*p*-Value
*N*	14	34	8	n/a
Age (years)	47.1 ± 24.2	46.6 ± 17.3	36.3 ± 30.8	0.554
Gender (male/female)	6/8	24/10	3/3	0.168
Letenneur type (I/II/III)	5/2/5	9/16/9	2/1/3	0.297
Follow-up (months)	19.1 ± 24.1	25.6 ± 29.7	52.5 ± 10.6	0.130

The mean follow-up was 19 months (range, 2–108 months). High-energy trauma mechanism was the most common cause of the sustained coronal plane shear fracture (*n* = 46 (82.1%)).

Preoperative radiographic analysis included conventional radiographs in 42 (75%) patients, followed by native computed tomography (CT) in 36 (64.3%) patients ± 3D reconstruction in 30 (53.6%). Magnetic resonance imaging (MRI) was performed in only five (8.9%) cases. MRI revealed bony avulsion of the medial collateral ligament in two cases, meniscal and chondral injury in one case and no concomitant soft tissue injury in the other two cases.

Nine (16%) patients had a medial Hoffa fracture, whilst in 47 (83%), the lateral femoral condyle was affected. Letenneur type II fractures were the most common, followed by Letenneur type III fractures. Most fractures (34; 60.7%) were addressed with screws only, eight (14.3%) patients were treated with a plate osteosynthesis, and in 14 (25%) subjects, a combination of plate and screws was used. Screw fixation was performed using 3.5 mm cortical screws placed 90° to the fracture line (antero-posterior more often than postero-anterior placement) and inserted preferably from the non-cartilage surfaced area or the screw heads were countersunk respectively. The use of biodegradable screws was rare and reserved for replacement of impressed fragment to restore the articular surface.

The surgical approach depended on the type of fracture and whether medial or lateral femoral condyle was involved. The direct lateral approach was favoured, followed by lateral parapatellar arthrotomies. The direct medial approach or medial parapatellar arthrotomies were performed in six (10.7%) and five (8.9%) cases, respectively. A combined approach (lateral subvastus and parapatellar arthrotomy) was performed in three cases. Lateral epicondyle osteotomy was necessary in three (5.4%) cases, and a median knee approach was made in two cases (3.6%). Arthroscopic-assisted fracture reduction and fixation was reported in one case (1.8%).

Intraoperative findings of additional knee injuries revealed significantly more often cartilage fissuring in Letenneur type III fractures (*p* = 0.042), whilst in Letenneur type I fractures, significantly, more often meniscal and full-thickness cartilage injuries (*p* = 0.027 and *p* = 0.007) were seen. Overall, any cartilage and meniscus injury were observed in 19.6% (*n* = 7) and 5.4% (*n* = 4) of the cases. A clinical case demonstrates concomitant soft tissue injuries in a Letenneur Typ II fracture and its complexity of surgical treatment (Fig. 2).

Postoperative management included partial weight-bearing in all surgically treated patients. Range of motion was inconsistently restricted depending on the type of fracture and surgical fixation method.

**Fig. 1:**
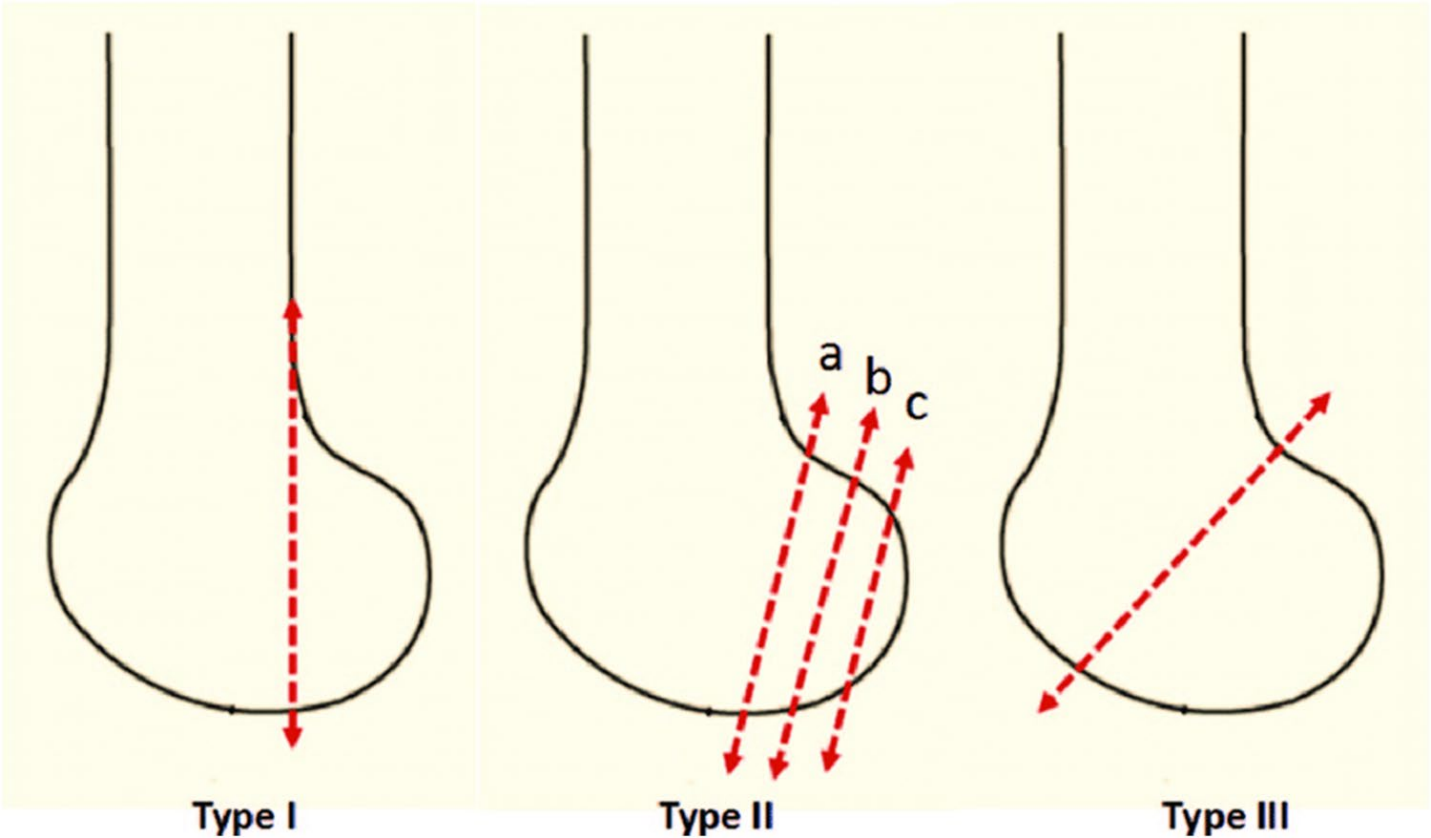
Descriptive image of the Letenneur classification [21]

Postoperative knee flexion at final follow-up was significantly better in patients who underwent isolated screw fixation compared to hybrid fixation techniques (*p* = 0.032) (Fig. 1). Letenneur type III fractures were significantly more likely to have an extension deficit of > 5–10° (*p* = 0.040) (Fig. 3).

**Fig. 2:**
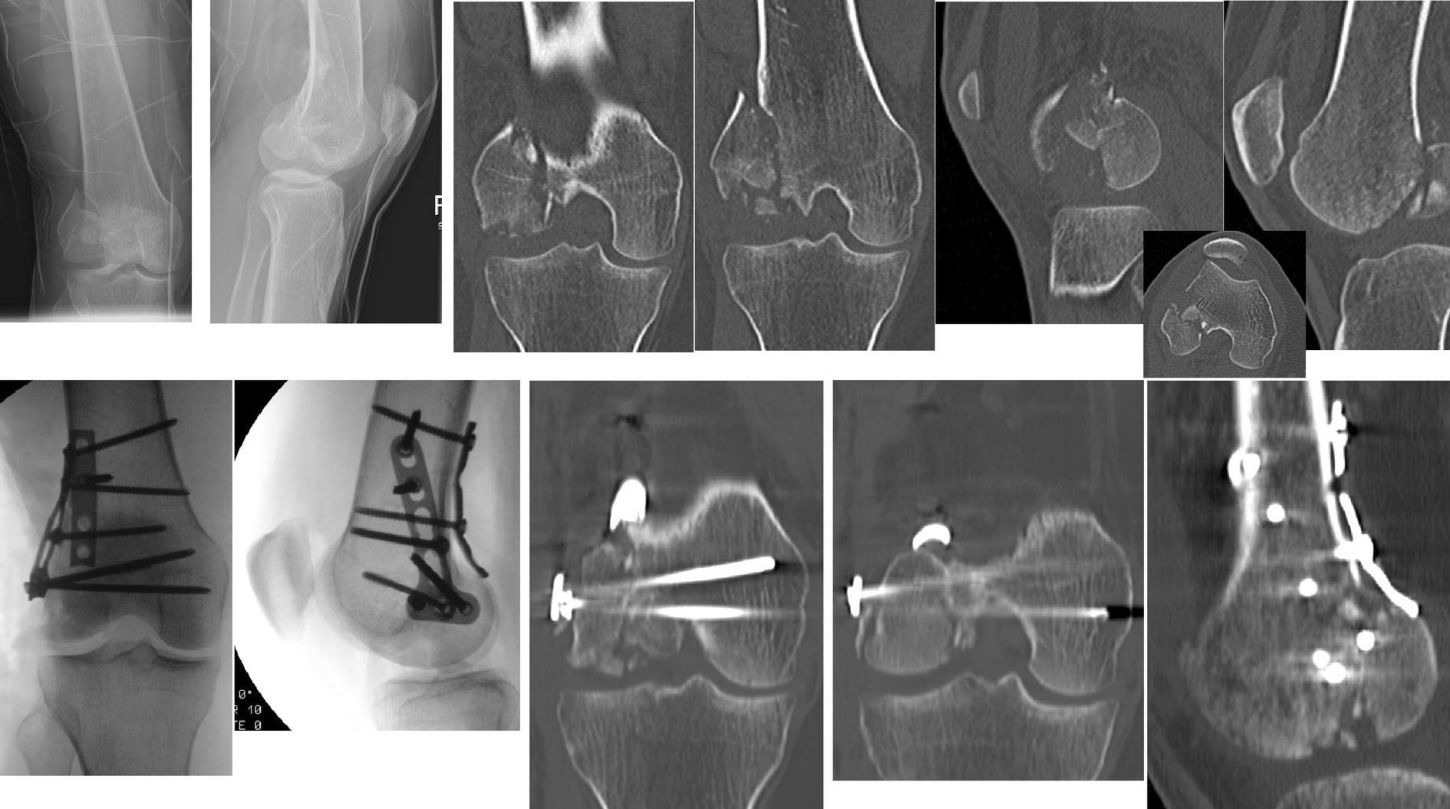
A 36-year-old female patient sustained a lateral Letenneur type II fracture. Surgical approach was performed using an osteotomy of the lateral epicondyle. The central impression fragment was replaced, and the defect was filled with allogenic spongiosa bone. Fracture fixation was achieved with one biodegradable compression screw, one freely inserted 3.5 mm cortical screw, a 3.5 mm T-shaped plate on the lateral side and a 4-hole reconstruction plate placed dorsally. The additive soft tissue injuries were managed with a direct suture of the lateral meniscus and minced cartilage technique for the sustained chondral lesion

**Fig. 3:**
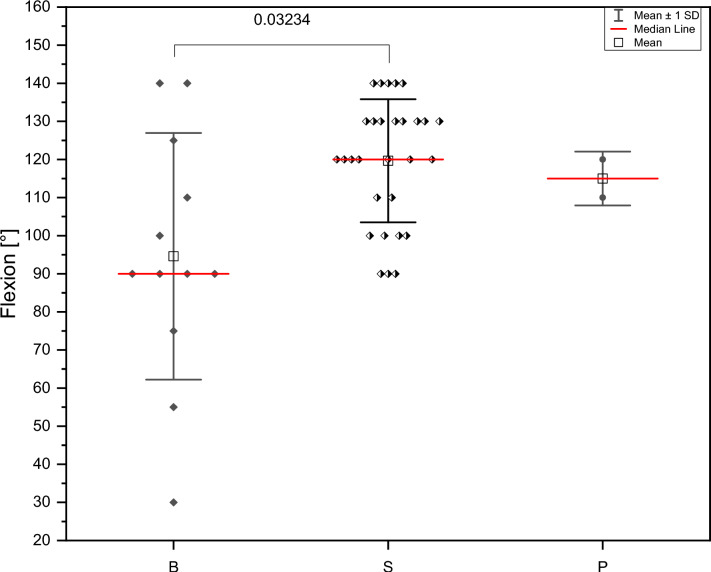
Range-of-motion at final follow-up comparing different fixation methods (B = Both = combined fixation technique plate and screw; S = Screw = screw fixation; P = Plate)

The overall postoperative complication rate was significantly higher in Letenneur type I fractures (*p* = 0.018). Functional outcomes for ranges-of-motion and postoperative complications in relation to the sustained Letenneur fracture type are shown in Table 2.

**Table 2:** Functional results for range-of-motion and complications in relation to the fracture type

Letenneur classification	1	2	3	*p*-Value
*n*	16	19	17	
Intraoperative findings^a^ (total patients yes/no) (%)	7/9 (77%)	8/11 (72%)	8/9 (88%)	0.95532
Chondromalacia Grade 0/1/2/3/4	16/0/0/0/0	14/3/0/2/0	14/0/0/1/2	0.94728
Cartilage fissuring (yes/no) (%)	0/16 (0%)	2/17 (12%)	5/12 (42%)	0.04196
Osteochondral flakes (yes/no) (%)	6/10 (60%)	3/16 (19%)	2/15 (13%)	0.15035
Meniscus tear/ACL/PCL tear/no	1/1/14	0/0/19	0/0/17	0.96330
Total findings	8	10	10	n/a
Overall complications (patients: yes/no) (%)	11/5	6/13	4/13	0.01864
Impaired wound healing	2/14 (14%)	1/18 (5%)	0/17 (0%)	0.30375
Surgical-site infection	2/14 (14%)	2/17 (12%)	0/17 (0%)	0.30375
Fixation failure	1/15 (6%)	1/18 (5%)	1/16 (6%)	0.99196
Delayed bone healing	1/15 (6%)	2/17 (12%)	1/16 (6%)	0.84357
Non-union	2/14 (14%)	2/17 (12%)	0/17 (0%)	0.34089
Osteonecrosis	0/16 (0%)	0/19 (0%)	0/17 (0%)	> 0.9999
Revision surgery	5/11 (45%)	2/17 (12%)	1/16 (6%)	0.09939
Instability	2/14 (14%)	1/18 (5%)	3/14 (21%)	0.50434
Meniscus injury	3/13 (23%)	0/19 (0%)	0/17 (0%)	0.02783
Cartilage injury	4/12 (33%)	0/19 (0%)	0/17 (0%)	0.00764
Intraarticular step formation	2/14 (14%)	1/18 (5%)	1/16 (6%)	0.68483
Axial malalignment	1/15 (6%)	0/19 (0%)	0/17 (0%)	0.31757
Implant removal	2/14 (14%)	1/18 (5%)	1/16 (6%)	0.68483
Post-traumatic osteoarthrosis	4/12 (33%)	1/18 (5%)	2/15 13%)	0.22686
Total complications (one patient could have more than 1 complication)	29	14	10	n/a
Follow-up [months]	31.4 ± 32.2	29.3 ± 32.0	12.2 ± 13.5	0.11484
Postoperative flexion restriction (yes/no)	13/3	14/5	13/4	0.86806
ROM according to neutral-0-method	13/0/1	12/2/1	13/0/0	
Extension (0°/5°/10°)	13/1/0	13/0/2	9/4/0	0.30616
Extension deficit (0°/5°/10°)	106.1° ± 30.1°	118.0° ± 20.1°	111.9° ± 21.6°	0.04007
Flexion (Mean ± SD)				0.68525

^a^Depending on the findings surgical managed complication

## Discussion

The main findings of the study were that most of the fractures were seen in the lateral condyle and classified as type II according to the Letenneur classification and screw fixation was the preferred surgical technique. Although MRI was only performed in five subjects, additional soft tissue injuries were frequently observed, especially in Letenneur type I and III fractures. Injuries to the menisci were managed with inside out suture technique or partial resection in case of vast destruction. Chondral lesions were addressed depending on the defect size with either microfracturing or minced cartilage technique. A significant extension deficit of 5–10° was documented after Letenneur type II fractures (*p* = 0.040), and knee flexion was significantly better after screw fixation compared to other surgical techniques (*p* = 0.032). The postoperatively limited extension following Letenneur type II injuries, which were primarily treated with plate osteosynthesis, is most likely due to postoperative scarring or arthrofibrosis rather than the postoperative rehabilitation protocol. Other postoperative complications, such as loss of fixation and delayed bone healing, were found in one patient in each group according to Letenneur type I–III fracture classification. Non-union was seen in two patients with Letenneur type I or II fractures. These complications required surgical revision and might have led to an axial malalignment postoperatively. The documented intraarticular step formation as well as the sustained cartilage injuries may explain the observed posttraumatic osteoarthritis during follow-up.

Identification of coronal plane fractures can be difficult, and no single radiographic examination can be universally recommended [3]. Whilst in plain radiographs Hoffa fractures are frequently missed, oblique radiographs and CT scans may facilitate detection of these coronal plane fractures [22–24]. These recommendations are in line with the radiographic examinations performed in the present study, with conventional radiographs performed in 75% and CT scans in 100% of patients.

The surgical approach to address Hoffa fractures depends on the location of the injury and the presence or absence of a comminution zone [25]. The main priority in any type of intra-articular fractures is to restore the articular surface. Many studies have reported the use of a lateral or a medial parapatellar approach to access the anterior femur [22, 26]. With these approaches, antero-posterior screw placement can easily be achieved. However, anatomic fracture reduction might be insufficient as the posterior femoral condyles cannot be properly visualised [27]. In a multicenter study of 18 patients, an antero-medial or antero-lateral approach was used in 78%, and a postero-lateral or postero-medial approach was applied in 22%. Fracture fixation was achieved with either antero-posterior or postero-anterior screw insertion [28]. In our series, the direct lateral approach was predominantly used (36%), followed by lateral parapatellar arthrotomies (30%). A direct medial approach to the knee was performed in 10%.

Surgical fixation of Hoffa fractures depends on the morphology of the fracture. Letenneur type II and III fractures are commonly fixed with 3.5 mm lag screws [27]. Fixation with two or more screws can prevent fragment rotation and therefore postoperative dislocation [29]. Antero-posterior screw insertion is associated with less soft tissue dissection and does not carry the risk of damaging the posterior neurovascular structures [30]. However, biomechanically, postero-anterior screws seem to have superior stability compared to antero-posterior screw insertion [4, 31]. In patients with a higher body mass index or poor compliance, osteoporosis, metaphyseal fracture extension or comminuted Hoffa fractures, a combined fixation technique using screws and a plate is recommended [19, 32]. Several biomechanical studies have shown that such combined techniques reduce the likelihood of fracture displacement and achieve good results [18, 33]. In our study, plate and screw fixation was associated with fixation failure and subsequent revision surgery (*p* = 0.008). Delayed osseous healing or non-union was seen in four cases after screw fixation and in two cases after combined or a screw fixation. In relation to the Letenneur classification, we found delayed bone healing in one case in Letenneur type I, in two cases in Letenneur type II and one following Letenneur type III fractures. Non-union was seen in four cases, two in Letenneur type I and two in Letenneur type II fractures.

A recent meta-analysis reviewed Hoffa fractures with associated injuries around the knee joint [34]. In this meta-analysis, 12 patients were identified with associated injuries and of these, 50% had an injury to the patella or the extensor mechanism. These findings are in contrast with our observations as no injuries to the extensor mechanism or patella was observed. However, in the current study, 11 (19.6%) cartilage injuries (including fissuring and full-thickness defects) and three (5.4%) meniscus tears were detected.

In a cohort study of 22 patients with surgically treated Hoffa fractures, postoperative complications were stiffness and pain in 4%, collateral laxity and progression of arthritis in each 4% after a follow-up of 12 months [35]. We found a progression or onset of posttraumatic osteoarthritis in 7 of 45 reviewed patients, which accounts for 15.5%. Collateral laxity was documented in 6 of 46 reviewed patients (13%).

### Limitations

There are several limitations of the present study that need to be considered. The retrospective nature of the study limits the precision of the data. The individual surgical treatments and the choice of implants were at the discretion of the operating surgeon. The multicenter nature of the study may limit the overall power and the interpretation of the individual data. Follow-up was relatively short with only 19 months. Furthermore, as Hoffa fractures are rare, the number of included subjects limits the power of the study significantly. However, several strengths of the study should be highlighted. This study collected multicenter data on current fixation methods for Hoffa fractures and analysed additional knee injuries and postoperative complications. To our knowledge, the number of patients included is relatively high considering current literature. Finally, knowledge on the likelihood for concomitant soft tissue injury provides valuable information for surgeons considering preoperative planning including additional MRI, surgical treatment with optional additional arthroscopy for verification of concomitant soft tissue injuries and postoperative aftercare.

## Conclusion

The present study showed that Hoffa fractures are predominantly caused by a high-energy mechanism and that mainly the lateral femoral condyle is affected. Meniscal and chondral lesions are commonly seen in Hoffa fractures and may need additional treatment. Screw fixation may have a better clinical outcome and remains the preferred method of fixation in simple fractures without a relevant comminution zone.

## Data Availability

All data generated or analyzed during this study are included in this published article.
